# Efficient Approaches to the Design of Six-Membered Polyazacyclic Compounds—Part 1: Aromatic Frameworks

**DOI:** 10.3390/molecules30153264

**Published:** 2025-08-04

**Authors:** Elena A. Gyrgenova, Yuliya Y. Titova, Andrey V. Ivanov

**Affiliations:** A. E. Favorsky Irkutsk Institute of Chemistry, Siberian Branch of the Russian Academy of Sciences, Favorsky Str. 1, 664033 Irkutsk, Russiaivanov@irioch.irk.ru (A.V.I.)

**Keywords:** azaheterocyclic, diazine, triazine, tetrazine

## Abstract

This review summarises the possible applications and basic methodologies for the synthesis of six-membered polyazo heterocycles, namely, diazines, triazines, and tetrazines. The time period covered by the analysed works ranges from the beginning of the 20th century to the present day. This period was chosen because it was during this time that synthetic chemistry, as defined by physicochemical research methods, became capable of solving such complex problems as efficiently as possible. The first part of the review describes the applications of polyazo heterocyclic compounds, whose frameworks are found in the composition of drugs, dyes, and functional molecules for materials chemistry, as well as in a wide variety of natural compounds and their synthetic analogues. The review also systematises the methods for assembling six-membered aromatic polyazo heterocycles, including intramolecular and sequential cyclisation, which determine the possible structural and functional diversity based on the presence and arrangement of nitrogen atoms and the position of the corresponding substituents.

## 1. Introduction

Nitrogen is one of the essential elements which, being a part of natural compounds, provides functional diversity. A special place among nitrogen-containing compounds is occupied by polyazo heterocycles, which can undoubtedly be called an indispensable attribute of life. Such heterocycles provide oxygen transport in organisms (e.g., chlorophyll and hemoglobin) and transfer hereditary information (e.g., the nitrogenous bases of DNA and RNA), which makes reproduction and evolution of life possible.

A close look at natural molecules with azaheterocyclic scaffolds (apart from the aforementioned compounds, these include vast libraries of alkaloids that regulate activity of virtually all types of organism), drugs, dyes, and advanced materials reveals that such systems are mainly represented by polyazo annulated heterocycles. In addition to their use in drug development and advanced technology materials, *N*-heterocyclic compounds are also in demand in synthetic chemistry due to their reactivity. Therefore, it is not surprising that the development of modern approaches to the synthesis of polyazo annulated heterocycles is an urgent task for the global synthetic community.

The chemistry of polyazocyclic compounds has recently been the subject of active development. The synthesis of diazine, triazine, and tetrazine derivatives allows the discovery of new drugs and preparation of high-energy systems. Nitrogen-containing heterocycles are important because they often form the basis of many pharmaceutically and agrochemically active compounds. The search for new materials with high energy density is being conducted among hybrid compounds, i.e., substances that incorporate different nitrogen-containing heterocycles. Researchers believe that combining different heterocycles into one molecule endows it with new, useful properties. Polyazo heterocyclic compounds constitute an important area of organic chemistry. Nitrogen heterocycles have been found to mimic various endogenous metabolites and natural products, emphasising their key role in the development of modern drugs. They have diverse applications and are predominantly used as pharmaceuticals, corrosion inhibitors, polymers, agrochemicals, dyes, and developers. Their catalytic behaviour makes these compounds notable precursors in the synthesis of various important organic compounds.

The present literature review summarises works that describe polyazo compounds based on six-membered aromatic heterocycles and their applications and methods of preparation. Given the diversity and volume of existing literature data, the proposed systematisation seems to be the most effective method of presentation.

## 2. Application of Six-Membered Polyazocyclic Compounds

### 2.1. Diazines

**Figure 1:**
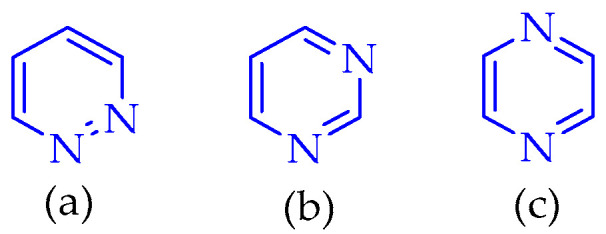
Six-membered heterocycles with two nitrogen atoms: (**a**) pyridazine, (**b**) pyrimidine, and (**c**) pyrazine.

Diazines are six-membered heterocyclic compounds containing two nitrogen atoms: pyridazines, pyrimidines, and pyrazines (Figure 1). This section summarises the literature data on their applications and methods of preparation. The diverse biological activities of diazines are well documented in research papers and reviews [1,2,3,4,5,6,7].

Due to their wide range of biological properties, including antiviral [8,9], antifungal [10], antimicrobial [11,12,13,14,15], anticancer [16], antitubercular [17], anti-inflammatory [18], antibacterial [19,20,21], antidiabetic [22], and others [23,24,25], pyridazine analogues have long been of interest for both medicine and agriculture [26,27,28,29].

**Figure 2:**
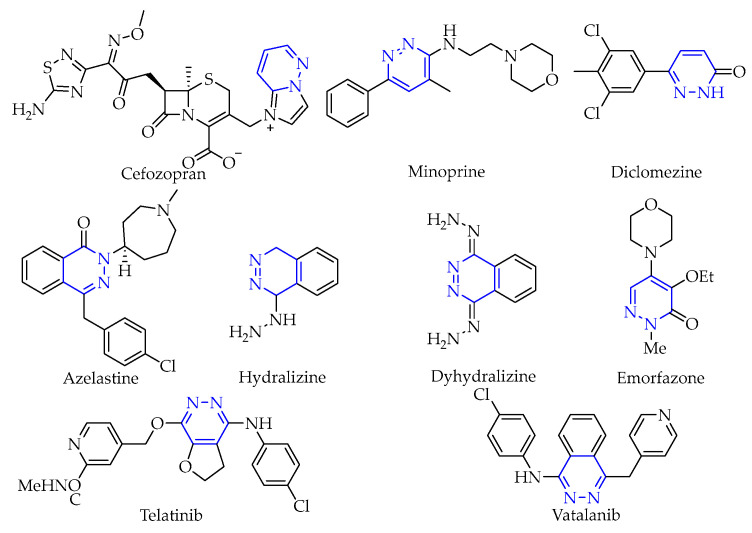
Examples of registered medicinal products comprising a pyridazine moiety.

The pyridazine structural motif is a popular pharmacophore and is found in some drugs, such as the fourth-generation cephalosporin antibiotic Cefozopran and the psychotropic drug Minaprin. Optivar (azelastine hydrochloride) is indicated for the treatment of itchy eyes associated with allergic conjunctivitis [30]. Aprésolien (hydralazine) and its analogue Cadralazine are antihypertensive vasodilators [31]. Digihydralazine^®^ is used to treat hypertension [32] (Figure 2).

Pyridazine core is also met in several herbicides, including Credazine, Pyridafol, and Pyridate. It is incorporated in metal complex ligands that can be applied as active electrocatalysts for proton reduction [33,34,35]. The electrochemical and photophysical properties of new organic light-emitting diodes containing the pyridazine acceptor part have been studied [36]. [1,2,5]-Thiadiazolo [3,4-d]pyridazines are used as internal acceptors for sensitisers [37]. Pyridazine scaffold is of great importance in optoelectronics because of its highly fluorescent properties, and it can be employed as sensors, biosensors, electroluminescent materials, lasers, and semiconductor devices [38,39,40,41,42].

Some pyrimidine analogues exhibit a variety of properties, including antiviral [43], antibacterial [44,45,46], anticancer [47], antidiabetic [48], antiparasitic [49], anticonvulsant [50], and others [51]. They also act as antidepressants [52] and analgesics [53].

**Figure 3:**
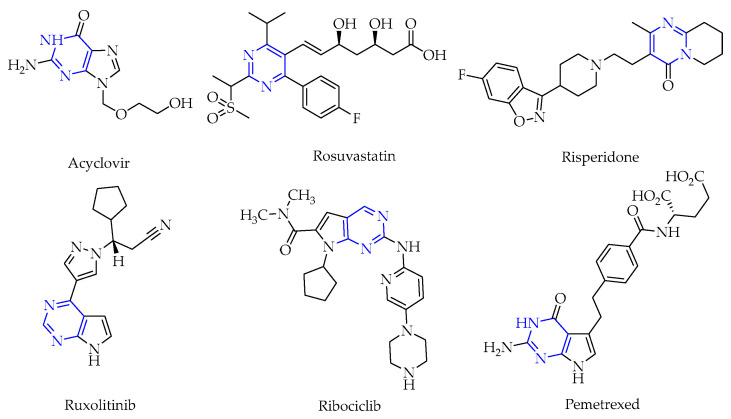
Examples of registered medicinal products comprising a pyrimidine fragment.

The best-known drug containing a pyrimidine fragment is Acyclovir [54,55], which was the first effective antiviral agent to be discovered. Valacyclovir, which is also available on the market under the brand name Vitrax, was derived from Acyclovir [56]. Rosuvastatin (Crestor) is a fourth-generation hypolipidaemic drug that inhibits cholesterol synthesis [57]. Allopurinol (Zyloprim) is mainly used to treat hyperuricaemia (elevated uric acid levels in the blood) and its complications [58]. Risperidone is effective in therapy of Alzheimer’s disease and substance abuse disorders [59]. Pyrrolopyrimidines are efficient antitumour drugs. For instance, Ruxolitinib is indicated for the treatment of myelofibrosis [60], Ribociclib is recommended against breast cancer [61], and Pemetrexed is used in therapy of non-small cell lung cancer [62,63]. Tubercidin inhibits the growth of certain bacterial strains [64]. Toyocamycin is a well-known antitumour antibiotic [65,66] that is active against L1210 leukaemia, P338 leukaemia, and Lewis lung carcinoma. It has also been tested in clinical trials for use against colon cancer, gallbladder cancer, and acute myelogenous leukaemia in humans [67] (Figure 3).

Pyrimidines are used as building blocks for aromatic [68] and partially aromatic compounds [69,70], in biorthogonal reactions [71], as flavouring agents [72], luminescent materials [73], and in combination with metal ions for design of highly structured molecular architectures [74]. Pyrimidine-based oligomers were investigated for their optical absorption and emission properties and for utilisation as a pH indicator [75]. Photoreactive surfaces were prepared by attaching pyrimidine molecules to planar gold substrates to form films [76]. It was shown that a series of carbene systems of palladium ligands was effective in the activation of methane and in the Mizoroki–Heck reaction [77].

Pyrazines are known to exhibit antitumour [78], antibiotic, anticonvulsant, antitubercular, and other activities [6,79,80,81,82,83,84]. They also possess antimicrobial and anti-inflammatory properties [85].

**Figure 4:**
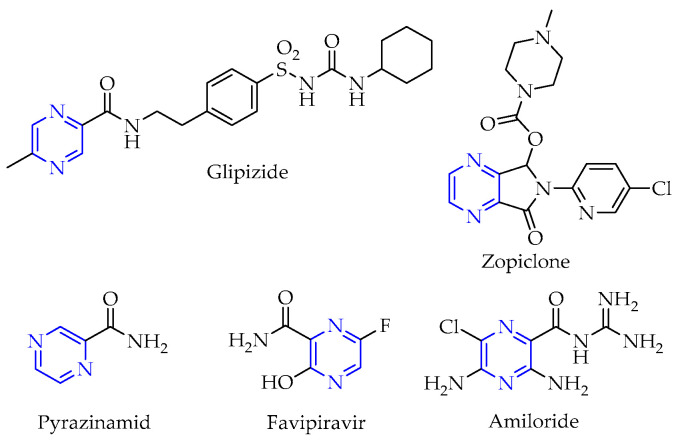
Examples of registered medicinal products comprising a pyrazine moiety.

Pyrazine derivatives are pharmacologically active. Pyrazinamide is a well-known antitubercular agent [86]. Glipizide and Sitagliptin (Januvia) are efficient against diabetes [87], and Amiloride is used as a diuretic [88]. Bortezomib and Oltipraz are indicated for therapy of cancer [89]. Sildenafil (Viagra) is used to treat erectile dysfunction, pulmonary arterial hypertension, and other diseases [90]. Studies on the efficacy of this drug in Alzheimer’s disease are also known [91]. Doxazosin (Cardura) is a non-selective alpha-1-adrenergic antagonist (alpha-adrenoblocker) employed in the therapy of arterial hypertension and benign prostatic hypertrophy [92]. Levofloxacin (Levaquin) is the antibiotic against bacterial infections [93]. It was also approved for the management of *H. pylori*-associated out-of-hospital pneumonia [94]. Ciprofloxacin is a broad-spectrum antimicrobial agent belonging to the fluoroquinolone group of antimicrobials [95,96]. Ketoconazole (Nizoral) is primarily employed in the treatment of fungal skin infections [97]. Trazodone is a drug efficient against depressive and anxiety disorders [98] and it is also used in the treatment of insomnia [99]. Aripiprazole (Abilify) is an atypical antipsychotic medication that can be employed for the treatment of Tourette’s syndrome [100]. Hydroxyzine (Atarax) reduces central nervous system activity and is used as a sedative [101,102]. Zopiclone is a non-benzodiazepine sleep-inducing pharmaceutical agent used for the short-term treatment of insomnia [103]. Meclizine (Bonine) is used to treat vestibular disorders [104]. Some of these drugs are depicted in Figure 4.

Pyrazines are used in agriculture as insecticides [105]. Some pyrazine derivatives are employed as flavouring and colouring agents in food products [106]. Additionally, quinoxaline compounds were appraised for utilisation as bulk heterojunction solar cells [107] and characterised as functionalised *p*-conjugated dendrimers with electronic applications [108]. Moreover, different types of polypyrazine derivatives are also used in the polymer industry as conjugated polymers [109] and semiconductors [110]. *n*-Type polymeric semiconductors based on dithienylpyrazindiimide were reported [111]. The polyimides constructed from a pyrazine moiety and cured at a low temperature find application in the modern microelectronics [112]. The fused ring pyrazine core impacts the thermal, electronic, and optical properties, as well as the thin film morphology, of organic field-effect transistors [113]. The utilisation of pyrimidine and pyrazine bridges as a design strategy was demonstrated to enhance the performance of thermally activated organic light-emitting diodes with delayed fluorescence [114,115].

### 2.2. Triazines

**Figure 5:**
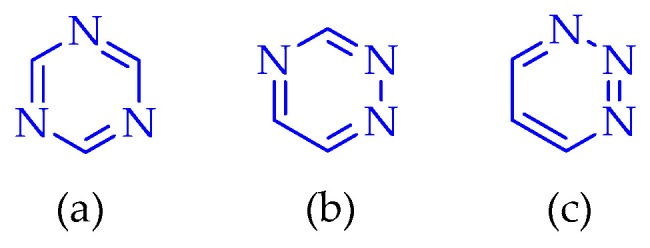
Six-membered heterocycles with three nitrogen atoms: (**a**) 1,3,5-triazine, (**b**) 1,2,4-triazine, and (**c**) 1,2,3-triazine.

Triazines represent an important class of six-membered aromatic heterocycles comprising three nitrogen atoms. There are three types of triazine regioisomers (Figure 5). 1,2,4-Triazines, also known as *a*-triazines, are an asymmetric subset of triazines. Among the three classes of triazines, 1,2,4-triazines are the most studied.

Biological activity of the compounds bearing a triazine structural motif is surveyed in reviews [116,117]. For instance, a series of 1,3,5-triazine derivatives exhibit antiviral activity that is higher than that of moroxidine hydrochloride [118]. The 1,2,4-triazine ring is frequently met in many natural or synthetic biologically active compounds with a wide variety of pharmacological properties. They are especially active as antitumour [119] and anti-AIDS agents [120], CRF receptor antagonists [121], antimicrobials, and anti-inflammatories [122]. A range of synthetic analogues of 1,2,3-triazine have been shown to possess biological activities, including antitumour [123], antimicrobial [124,125], antiviral [126], analgesic [127], anti-inflammatory [128], antihistamine [129], antiangiogenic [130], and antifungal [131].

**Figure 6:**
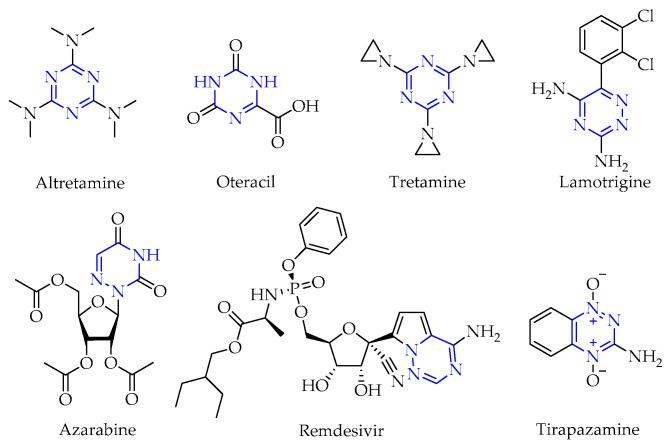
Examples of registered medicinal products comprising triazine moieties.

A plethora of drugs are based on triazine moieties. For example, Altretamine, an antitumour agent, is employed in the management of persistent or recurrent ovarian cancer [132,133,134,135]. Oteracil is a chemotherapeutic agent, which is used in combination with other pharmaceuticals for the treatment of advanced gastric cancer [136] and nasopharyngeal cancer [137]. Tretamine is an antitumour medicine that is utilised in the therapy for retinoblastoma [138]. Lamotrigine (Lamictal) is an antiepileptic drug to combat certain types of epilepsy and type I bipolar disorder [139]. Tirapazamine, also known as SR-4233, is an experimental anticancer drug [140], Azaribine is an antiviral agent [141], and Remdesivir (whose framework is pyrrolo[2,1-*f*][1,2,4]triazine) is an antiviral medicine [142] (Figure 6).

1,3,5-Triazines also play a prominent role in fluorescent chemosensor applications [143]. These compounds and their nitrogen-containing heterocyclic derivatives have key structural features with a wide range of photovoltaic characteristics and find application as UV absorbers, fluorescent probes, and phosphorescent organic light-emitting diodes [144,145,146]. Triazines are used as herbicides and pesticides, including atrazine, simazine, and cyanazine [147].

### 2.3. Tetrazines

**Figure 7:**
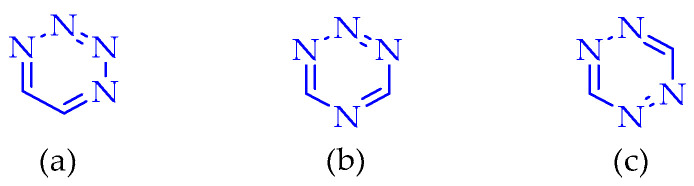
Types of six-membered heterocycles with four nitrogen atoms: (**a**) 1,2,3,4-tetrazine, (**b**) 1,2,3,5-tetrazine, and (**c**) 1,2,4,5-tetrazine.

Tetrazines represent an important and sought-after class of *N*-heterocycles that find applications in various fields of human activity among the three possible isomers (Figure 7).

Tetrazines are utilised as synthons in the synthesis of various alkaloids. For instance, lycorine possesses analgesic, antipyretic, and anti-inflammatory properties. The alkaloid hippadine exhibits cytotoxic activity against a number of tumour cells, whilst epibatidine is a potent analgesic [148]. 1,2,3,4-Tetrazines have anticancer and antimicrobial properties [149,150,151].

**Figure 8:**
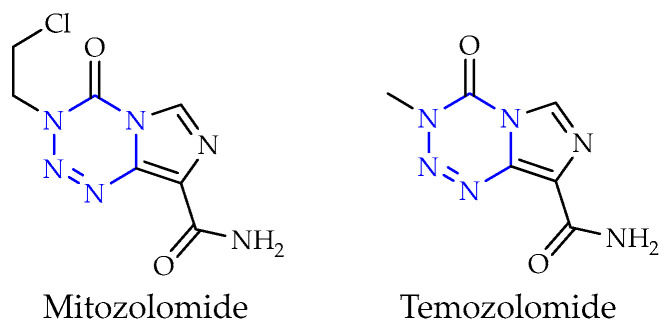
Examples of registered drugs comprising tetrazine moieties.

The antitumour drug mitozolomide was synthesised on the basis of imidazotetrazine. Further development of this pharmaceutical product was discontinued during phase II clinical trials, as the recommended dose proved was found to be too toxic, causing severe thrombocytopenia. Temozolomide proved to be less effective but less toxic than mitozolomide. It is currently marketed under the trade name Temodal (Temodar and Temomedac) and is used against malignant melanoma, mycosis fungoides, and brain tumours [152,153,154,155] (Figure 8).

In addition to their biological applications, tetrazines and their 3,6-disubstituted derivatives are of great interest to the field of coordination chemistry. They are characterised by unusual transfer of electron density and charge and the ability to coordinate metal centres through multiple mechanisms [156]. As an electron-deficient 4π-component, they participate in Diels–Alder reverse electron-transfer reactions [157]. Tetrazines are employed in the synthesis of energetic materials with a high nitrogen content [158,159]. Tetrazino-tetrazine-1,3,6,8-tetraoxide is considered to be one of the most potent energetic materials that has been synthesised to date [160]. Tetrazines conjugated to tetrazole are versatile bifunctional building blocks for the synthesis of linear oligoheterocycles [161]. Tetrazines linked to pyridine open access to substituted pyridazines, which are known for their high ability to coordinate metals [162]. Tetrazine-based metal–organic frameworks find applications in small molecule adsorption, sensing, and in the detection of organic functional molecules [163]. Tetrazines are utilised for the development of chromophoric nucleosides to produce coloured uridines ranging from purple to orange and red [164].

## 3. Synthesis of Six-Membered Polyazocyclic Compounds

The methods for assembly of six-member polyazo aromatic systems, namely, diazines, triazines, and tetrazins, as well as structures containing pyrrole condensed frameworks, starting with diazine structures, will be successively considered. Literature analysis shows that the main strategies for design of the above systems are (1) catalysis of transition metal complexes, both in the presence of specially introduced ligands and in the ligand-free version; (2) direct synthesis of polyazotic aromatic heterocycles, carried out in the presence of (i) strong organic bases, (ii) organocatalysts, under the influence of (iii) microwave or ultrasonic radiation, as well as (iv) electrochemically. Considering the significant role of pyrrolo-fused in pharmaceutical and material science, some methods for their production were considered. It should be noted that, in this part of the review, we will only discuss six-membered polynitrogen systems having aromaticity.

### 3.1. Synthesis of Diazine Derivatives

#### 3.1.1. Synthesis of Pyridazine Derivatives

**Scheme 1:**
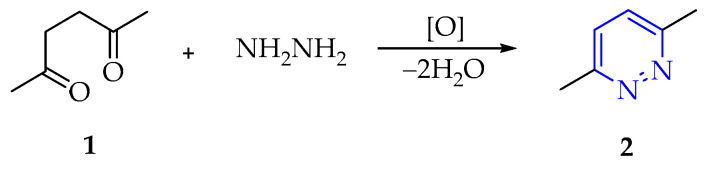
General method for the synthesis of pyridazines [170,171,172,173,174].

To date, a plethora of syntheses of chemically and pharmacologically valuable pyridazines have been developed. A variety of synthetic methodologies for the construction of pyridazine systems [165,166,167], including pyrrolopyridazines [168], were comprehensively documented in the literature. The pioneering data were obtained by Curtius [169] using the reaction of diethyl-1,2-diacetylsuccinate with hydrazine hydrate as an example. Consequently, an in-depth investigation of this reaction was initiated. The majority of general methods for the synthesis of pyridazines involves the condensation of 1,4-dicarbonyl compounds **2** with hydrazine **1** and its derivatives (Scheme 1) [170,171,172,173,174].

**Scheme 2:**
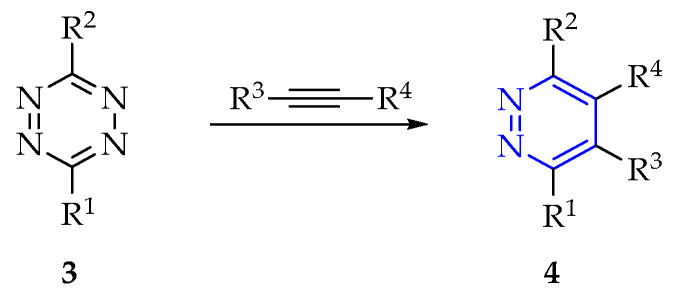
General method for the synthesis of **4** from **3** via the Diels–Alder reaction [176,177,178].

One of the general methods for the preparation of pyridazines has been known since 1972; the work of Meresz et al. [175] described the Diels–Alder reaction using 1,2,4,5-tetrazines as dienes and alkynes [176,177,178]. In a similar manner, 1,2,3-triazines were found to react with 1-propynylamines under neutral conditions [179]. Scheme 2 shows the general method for the synthesis of pyridazine derivatives **4** from tetrazines **3** via the Diels–Alder reaction.

**Scheme 3:**
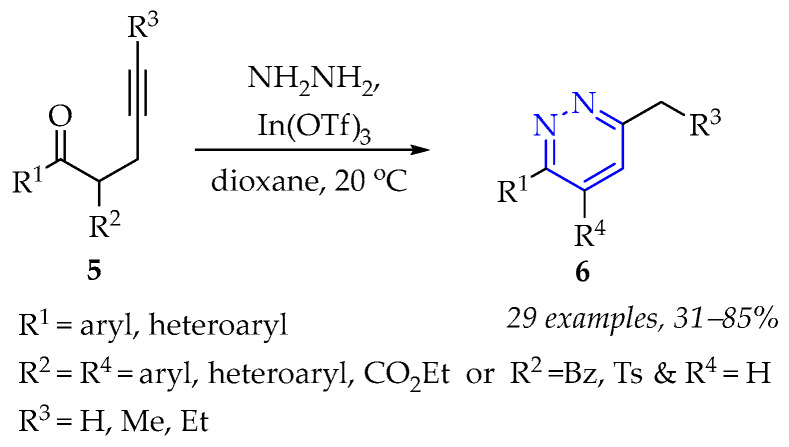
One-pot [4+2] cyclocondensation of substituted pyridazines [188].

Other methods for the synthesis of pyridazines include annelation of aldehyde hydrazones with *α,β*-unsaturated nitriles [180,181]; alkoxyallenes with 1,2-diaza-1,3-dienes [182]; ketene with *N*,*S*-acetals and *N*-tosylhydrazones [183]; cyclopropene derivatives with hydrazones [184]; catalytic three-component one-step annelation [185,186]; and multistep synthesis using the Diaz–Wittig reaction [187]. The cyclisation of alkynones **5** with indium(III) triflate-activated hydrazine afforded substituted pyrazines **6** (Scheme 3). When substrates containing a benzoyl or tosyl group were used as R^2^, this group was removed to form 4-substituted products [188].

**Scheme 4:**
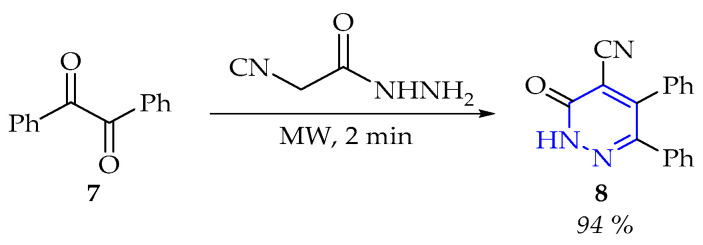
Pyridazine synthesised under green chemistry conditions using microwave irradiation [189].

Under microwave irradiation, dibenzyl **7** reacted with cyanoacetohydrazide to give 3-oxo-5,6-diphenyl-2,3-dihydro-pyridazine-4-carbonitrile **8** (Scheme 4). The microwave irradiation method gave better yield and needs short reaction time [189].

**Scheme 5:**
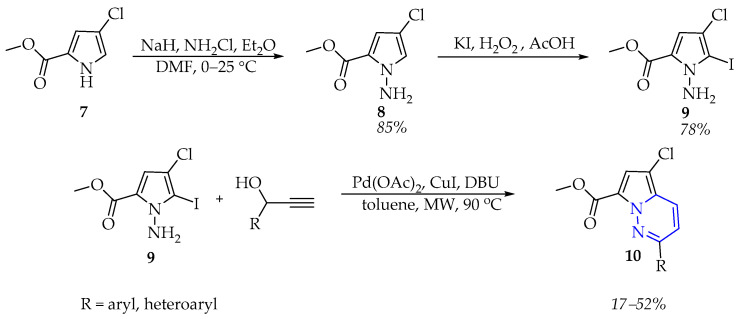
Domino-coupling–isomerisation–condensation reaction [196].

Pyridazines can be obtained by the recyclisation involving either the compression [190] or expansion of the diazepinone cycle [191]. Pyrrolopyridazine derivatives were first obtained by Flitsch and Krämer in 1968–1969; pyrrolo [1,2-*b*]pyridazines were synthesised from 1-aminopyrrol and its derivatives [192,193]. The synthesis of pyrrolo[1,2-*b*]pyridazine derivatives was reported in 1977 by Kuhla and Lombardino [194]. Pyridazines containing a pyrrole backbone are synthesised in a multistep manner [195]. The first step includes the functionalisation of the pyrrole backbone **7** at the nitrogen atom and then to the second position of the pyrrole ring **8**. After that, pyrrolo[1,2-*b*]pyridazines **10** were readily obtained via a domino-coupling–isomerisation–condensation reaction between **9** and the corresponding (hetero)arylpropargyl alcohols (Scheme 5) [196].

#### 3.1.2. Synthesis of Pyrimidine Derivatives

A number of reviews are devoted to the synthesis of pyrimidines [197,198]. For instance, the work by Pino-González surveys catalytic methods of preparation [199]. Pyrimidines are generally formed via a three-component reaction between aldehydes, ketones, and various amidines [200,201,202,203,204,205]. The use of a catalyst in the reaction allows the synthesis of substituted pyrimidines without organic solvents [206]. This section deals with other methods for the synthesis of pyrimidines.

**Scheme 6:**
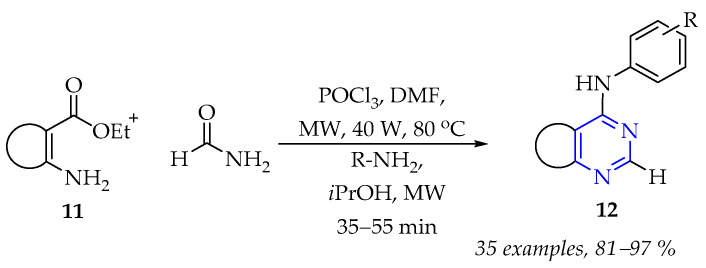
One-pot synthesis of condensed 2*H*-pyrimidin-4-amine libraries under microwave irradiation [207].

Pyrimidines **12** were obtained under microwave irradiation of a mixture of **11**, formamide, and phosphorus oxychloride (Scheme 6). The one-pot microwave-assisted synthetic protocol is high-yielding, ecofriendly, and eliminates intermediate workups [207].

Rostovskii [208] studied a novel photolysis of *α*-azidocinnamates, leading to the key intermediate 2*H*-azirines, which underwent the dimerisation ring-expansion cascade to produce fully substituted pyrimidines. For dimerisation involving 2*H*-azirine via a Pd/Ag cocatalysed intermolecular self-assembly, a wide variety of unsymmetrical tetra-arylsubstituted pyrimidines efficiently give a wide variety of moderate yields [209].

**Scheme 7:**
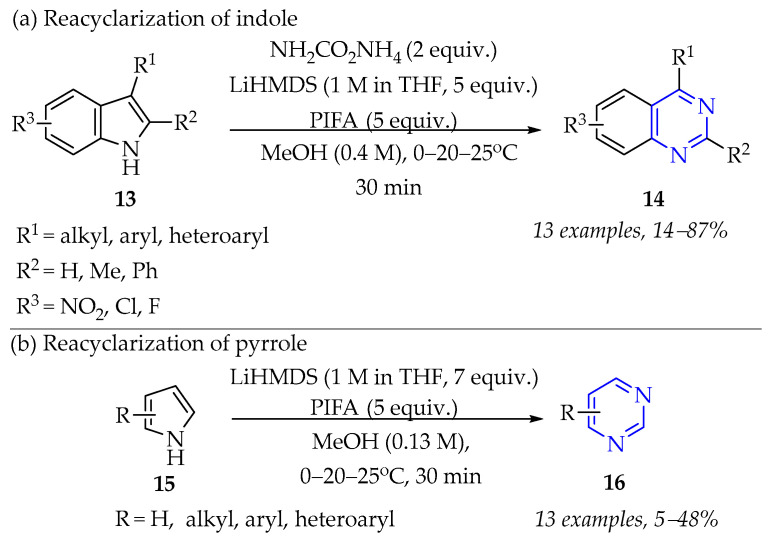
Skeletal editing of indoles and pyrroles: (**a**) recyclarization of indole, and (**b**) recyclarization of pyrrole [210].

Pyrimidines **14** were synthesised by the reacylisation of indoles **13**. The reaction was carried in LiHMDS under mild conditions (Scheme 7a). The same reaction was applicable to 1*H*-pyrroles **15** (Scheme 7b), which, in the presence of PIFA as an oxidising agent, gave compounds **16** [210].

**Scheme 8:**
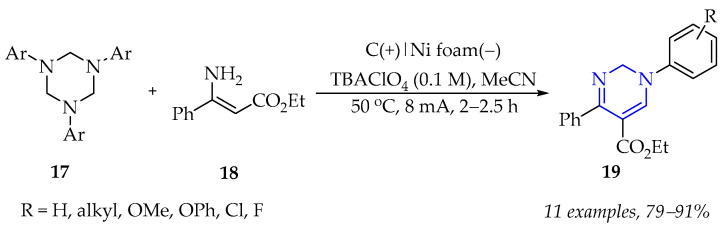
Electro-oxidative formal [3+3] annulation of 1,3,5-triazinane [211].

The electro-oxidative formal [3+3] annulation of 1,3,5-triazinane **17** with enamines **18** affording polysubstituted 1,2-dihydropyrimidines **19** was implemented (Scheme 8) [211]. It was noted that the reaction was carried out under mild conditions without transition metal compounds to give the target products in excellent yields.

**Scheme 9:**
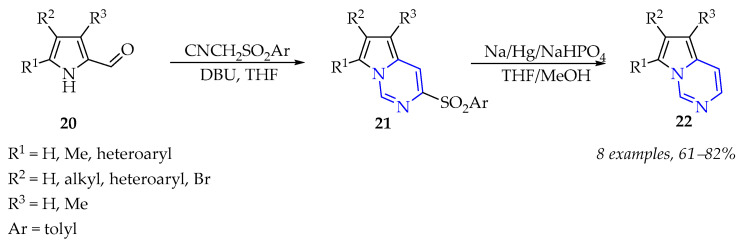
Cyclocondensation pyrroles with tosylmethylisocyanide [212].

In regard to pyrrolo[1,2-*a*]pyrimidines, methods for the preparation of these compounds can be categorised into three groups: syntheses starting with the preliminary formation of the pyrimidine part of the bicyclic system; syntheses starting with the preliminary formation of the pyrrole part; and syntheses from acyclic reagents only. A limited number of functional group transformations, such as esterification and nitrile hydrolysis, were documented for derivatives of the completely unsaturated pyrrolo[1,2-*a*]pyrimidine core. However, the reactions described for pyrrolo[1,2-*c*]pyrimidines fall into three main types: electrophilic attack, addition reactions, and cycle opening processes. It was disclosed [212] that the cyclocondensation of **20** with tosylmethylisocyanide followed by desulfonylation of 2-tosylpyrrolo[1,2-*c*]pyrimidines **21** with sodium amalgam afforded pyrrolo[1,2-*c*]pyrimidines **22** (Scheme 9).

**Scheme 10:**
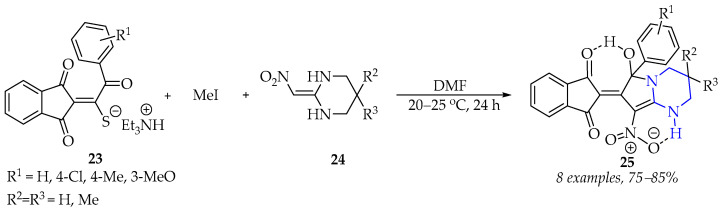
One-pot three-component synthesis of pyrrolo[1,2-*a*]pyrimidine [213].

A series of pyrrolo[1,2-*a*]pyrimidines **25** containing 1,3-indanedione skeleton (Scheme 10) was obtained by bicyclisation from triethylammonium thiolates **23**, methyl iodide, and **24** [213]. The unique property of this method is a simple procedure without using any metal catalysts or acid/base additives.

**Scheme 11:**
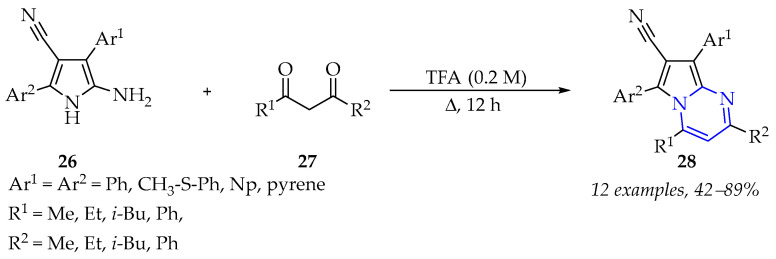
Construction of multisubstituted pyrrolo[1,2-*a*]pyrimidines [214].

A wide range of asymmetrically tetrasubstituted fused pyrrolo[1,2-*a*]pyrimidines **28** was obtained from *NH*-pyrroles **26** and diketones **27** and Scheme 11 shows the synthesis [214].

#### 3.1.3. Synthesis of Pyrazine Derivatives

There are many methods for the synthesis of pyrazine and its derivatives [215], some of which are among the oldest. Examples include the works of Staedel-Rügheimer [216], Gutknecht [114,217], and Gastaldi [218], which are still in use today.

**Scheme 12:**
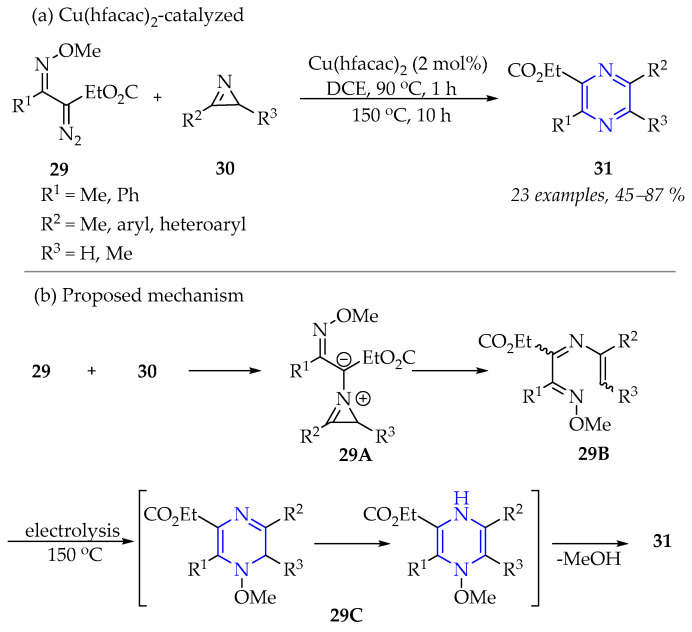
(**a**) Cu-catalyzed synthesis and proposed mechanism of unsymmetrical pyrazines, and (**b**) proposed mechanism [219].

The reaction of α-iminocarbenoids **29** with 2*H*-azirines **30** delivered asymmetrically substituted pyrazines **31** in good to high yields (Scheme 12a). The authors suggest that the reaction begins with the initial formation of the metallocarbene complex of **29** with **30** to give ylide **29A**, which is isomerised to 1,4-diazahexatriene **29B** (Scheme 12b). Further, 1,2-dihydropyrazine formed by electrocyclisation is rapidly converted to 1,4-dihydropyrazine **29C**, in which spontaneous cleavage of MeOH occurs to afford pyrazine **31**. This mechanism is consistent with the observed regiochemistry of pyrazines [219].

**Scheme 13:**
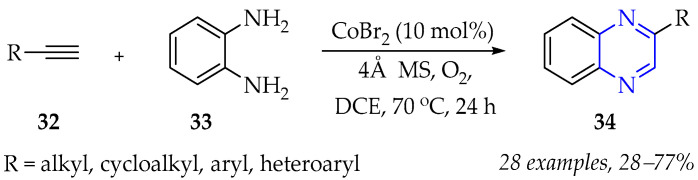
Co-catalyzed annulation of terminal alkynes and *o*-phenylenediamines [224].

Pyrazines can be synthesised via the dehydrogenating coupling of alcohols and amines catalysed by Ru [220], Ir [221], Co [222], Mg [223], and Pd [83] complexes. The reaction of alkynes **32** with diamine **33** under the action of divalent cobalt bromide led to a wide range of substituted pyrazines **34** (Scheme 13) [224].

The same reaction can proceed with DABCO as a catalyst, which activates sulphur to give the target products in more than 50% yields [225]. Also, a catalyst-free approach to the synthesis of pyrazine derivatives from sulfoxonium ylides and *o*-phenylenediamines with the participation of elemental sulphur was developed [226].

Pyrrolopyrazine derivatives have been studied for several decades [227,228]. They can be synthesised in various ways: from functionalised pyrazines or pyrroles and via a three-component reaction from acyclic compounds. Pyrrolo[1,2-*a*]pyrazines are also obtained by different methods, including the Pictet–Spengler reaction of isotryptamine and 2-(pyrrol-1-yl)ethanamine with trifluoromethylated carbonyl compounds and 2-perfluoroalkyl-substituted cyclic imines [229]; catalytic and thermal cycloisomerisations of propargyl-containing heterocycles [230]; and domino reaction of 2-pyrrolcarbaldehyde with vinylazides [231]. In addition, *N*-allylenylpyrrole-2-carbaldehydes [232,233] and *N*-propargylamino(pyrrolyl)enones [234] were employed as the starting compounds for the construction of such structures.

**Scheme 14:**
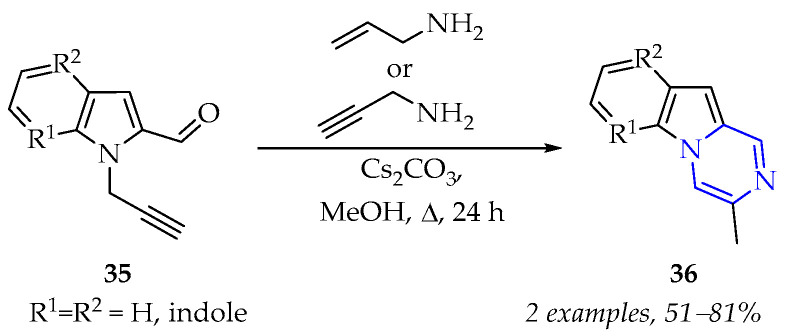
Cyclisation reactions of **35** with allylamine and propargylamine [235].

The reaction of *N*-propargylpyrrol-2-carbaldehyde **35** with various amines led to pyrrolo[1,2-*a*]pyrazines **36** (Scheme 14) [235].

**Scheme 15:**
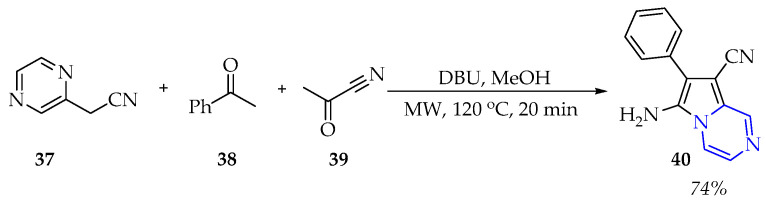
Synthesis via a Knoevenagel/[4+1] cycloaddition cascade [236].

A one-pot, three-component reaction between 2-methylene cyanoazaheterocycles **37**, aldehyde **38,** and acetylcyanide **39** afforded pyrrolo[1,2-*a*]pyrazine **40** (Scheme 15). The methodology involves the formation of a heterocyclic 1-aza-1,3-diene derived from a Knoevenagel condensation between an aldehyde and 2-methylene-cyano aza-heterocycles, followed by [4+1] cycloaddition of acetyl cyanide behaving as a non-classical isocyanide replacement [236].

### 3.2. Synthesis of Triazine Derivatives

The widespread use of triazines makes the development of efficient methods for their synthesis an important task. Methods for the preparation of 1,3,5-, 1,2,4-, and 1,2,3-triazines are described in detail below. Among the triazines described above, 1,2,3-triazines are the least studied because their ring system is considered the least stable, and their syntheses are limited.

#### 3.2.1. Synthesis of 1,3,5-Triazine Derivatives

The synthesis of 1,3,5-triazine compounds was first reported by A. Pinner in 1890 [237]. In the syntheses of 1,3,5-triazines, triamines or polyamines were used as nucleophiles, while the substrates included aldehydes and their derivatives [238].

**Scheme 16:**
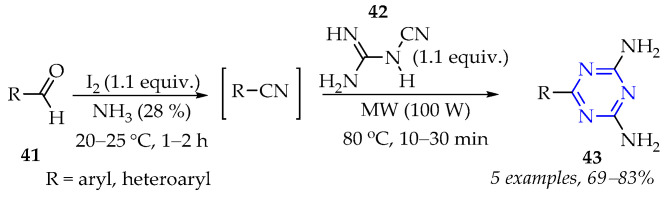
Microwave-assisted one-pot tandem reactions for direct conversion of primary aldehydes to triazines [241].

In 2002, Díaz-Ortiz et al. synthesised symmetrically substituted 1,3,5-triazines (30–84% yields) via the solvent-free cyclisation of nitrile reaction using Lewis acids on silicon oxide as catalysts under microwave radiation [239]. Later, this research team implemented the reaction of dicyandiamide with aromatic/aliphatic/heterocyclic nitriles under microwave irradiation in the presence of a base for 10–15 min to furnish 2,4-diamino-6-1,3,5-triazines in 52–92% yields [240]. Triazines **43** were also obtained in high yields by microwave irradiation-assisted reaction of primary aldehydes **41**, which were treated with iodine in ammonia water to give the intermediate nitriles, which were subjected to [2+3] cycloaddition with dicyandiamide **42** without isolation (Scheme 16) [241].

**Scheme 17:**
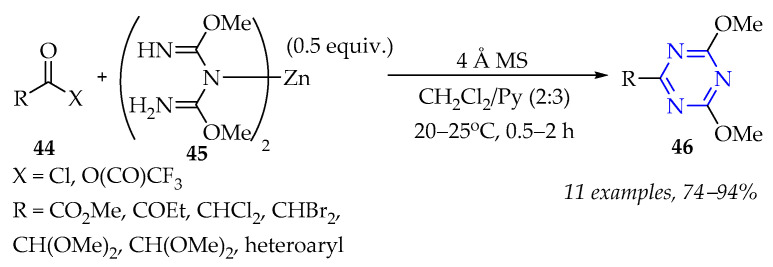
Condensation of acid chlorides or anhydrides **44** with salt **45** [242].

Acid chlorides or anhydrides **44** reacted with stoichiometric amounts of zinc dimethylimidodicarbonimidate zinc **45** to form 4,6-dimethoxy-1,3,5-triazines **46** in high yields (Scheme 17) [242].

**Scheme 18:**
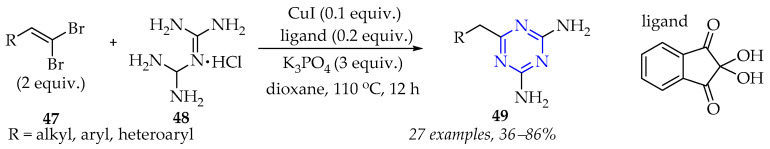
Copper-catalysed synthesis of substituted 2,4-diamino-1,3,5-triazines [243].

The CuI-catalysed reaction of 1,1-dibromoalkenes **47** and biguanides **48** gave rise to the substituted 2,4-diamino-1,3,5-triazines **49** (Scheme 18). The reaction tolerated alkyl-, heteroaryl-, or aryl-substituted 1,1-dibromoalkenes to afford the products in moderate to good yields [243].

**Scheme 19:**
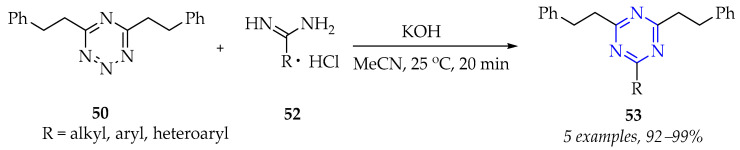
Reactivity of 1,2,3,5-tetrazines **50** [157].

The reactions of 1,2,3,5-tetrazine **50** with amidines **51** led to the formation of substituted 4,6-diphenethyl-1,3,5-triazine **52** (Scheme 19). All amidines examined, including aryl-, heteroaryl-, and alkyl amidines **51**, smoothly react with tetrazine **50** at ambient temperature to provide the 1,3,5-triazines **53** in uniformly high yields [157].

#### 3.2.2. Synthesis of 1,2,4-Triazine Derivatives

**Scheme 20:**
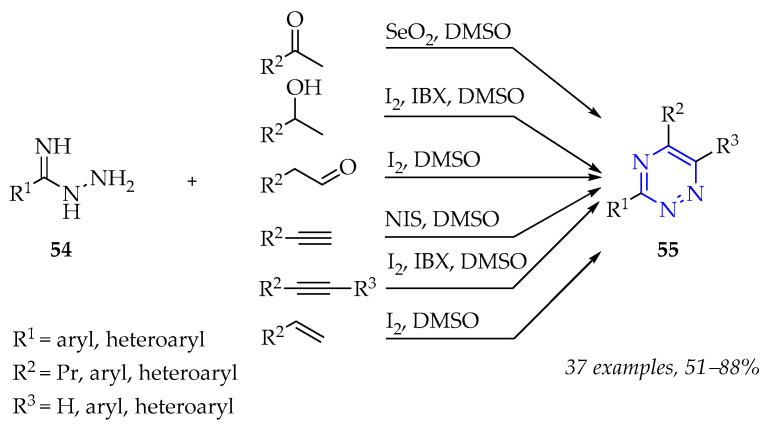
The transformations to construct 1,2,4-triazine derivatives [246].

Several reviews were devoted to the synthesis of 1,2,4-triazines [244,245]. For instance, 1,2,4-triazine derivatives **55** were prepared via simple and efficient [4+2] annulation reactions of imidohydrazides **54** with ketones, aldehydes, alkynes, secondary alcohols, and alkenes (Scheme 20) [246].

**Scheme 21:**
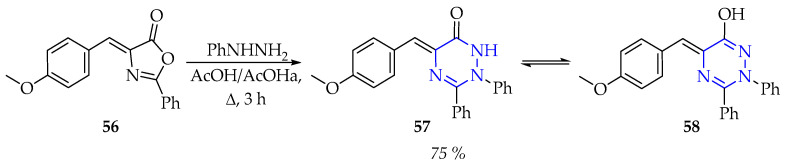
Synthesis of triazine derivative [247].

The reaction of oxazolone **56** with phenylhydrazine in the presence of acetic acid and sodium acetate delivered 1,2,4-triazine **57**. The lactam form **57** is thermodynamically more stable than the enolic form **58** (Scheme 21) [247].

**Scheme 22:**
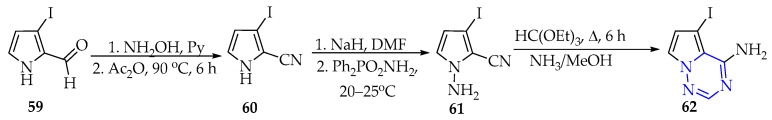
Multistep synthesis of pyrrolotriazine [248].

Pyrrolotriazine **62** was synthesised from 3-iodo-1*H*-pyrrole-2-carbaldehyde **59**, which was converted to pyrrole-2-carbonitrile **60** (Scheme 22). Electrophilic *N*-amination of compound **60** followed by cyclisation of the formed *N*-aminopyrrole **61** with triethylorthoformate gave pyrrolo[2,1-*f*][1,2,4]triazine **62** [248].

**Scheme 23:**
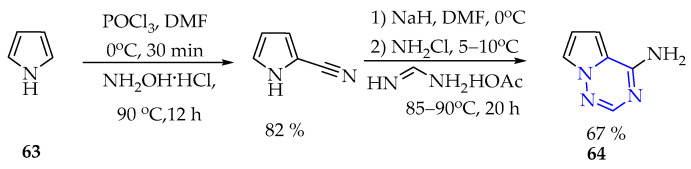
Two-step synthesis of pyrrolotriazine [249].

Pyrrolo[2,1-*f*][1,2,4]triazin-4-amine **64** was prepared by a two-step reaction from pyrrole **63** (Scheme 23) [249]. **64** is an important regulatory starting material in the production of the antiviral drug Remdesivir. This method is suitable for obtaining **64** in kilogram quantities.

#### 3.2.3. Synthesis of 1,2,3-Triazine Derivatives

**Scheme 24:**
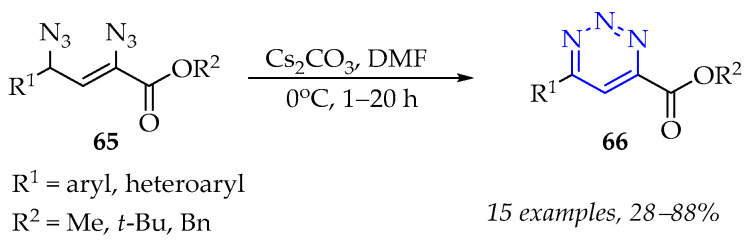
The base-mediated cyclisation of (*Z*)-2,4-diazido-2-alkenoates [250].

It was found that (*Z*)-4-aryl-2,4-diazido-2-alkenoates **65** underwent cyclisation under slightly alkaline conditions without transition metals or strong acids to furnish esters of 6-aryl-1,2,3-triazine-4-carboxylic acid **66** (Scheme 24) [250].

**Scheme 25:**
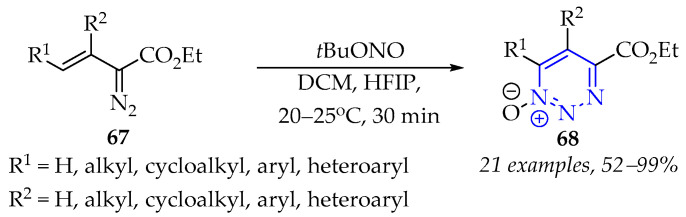
The [5+1] cycloaddition reaction for the directed synthesis of triazine-1-oxides [251].

The [5+1] cycloaddition reaction of vinyl diazo compounds **67** with *tert*-butyl nitrites was employed for the directed synthesis of 1,2,3-triazine-1-oxides **68**, which then underwent further functionalisation (Scheme 25) [251].

### 3.3. Synthesis of 1,2,3,4-Tetrazine Derivatives

1,2,4,5-tetrazine (*s*-tetrazine) is of particular interest due to its enhanced stability. The literature review revealed that 1,2,3,4- and 1,2,3,5-tetrazines have not been sufficiently investigated, and there are only a few examples of their synthesis. A selection of examples will be illustrated below.

#### 3.3.1. Synthesis of 1,2,3,4-Tetrazine Derivatives

The development of the experimental and theoretical chemistry of 1,2,3,4-tetrazines is comprehensively covered in the review [252]. The first compound with four neighbouring nitrogen atoms in a six-membered ring was obtained in 1972 by Nelsen and Fibiger in less than 5% yield [253].

**Scheme 26:**
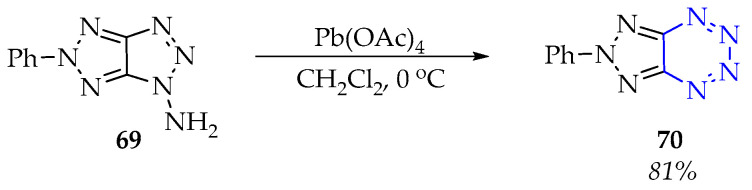
Synthesis of 1,2,3,4-tetrazine [254].

The oxidation of 1-amino-5-phenyl-1,2,3-triazolo[4,5-*d*]-1,2,3-triazole **69** with lead tetraacetate afforded 1,2,3,4-tetrazine **70** (Scheme 26) [254].

**Scheme 27:**
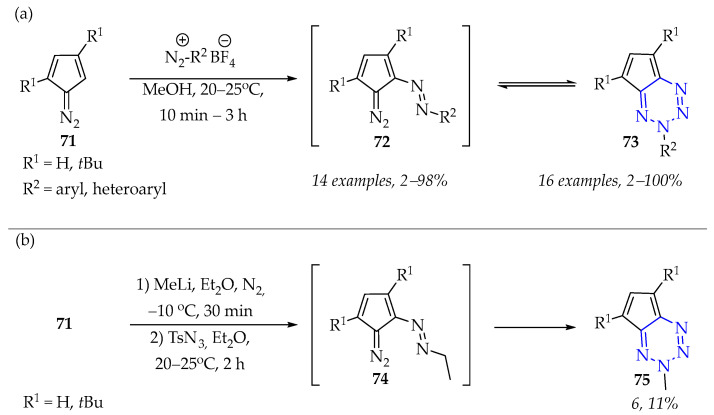
(**a**,**b**) Two different methods of synthesis of cyclopenta-annulated 1,2,3,4-tetrazines [255].

The conversion of diazocyclopentadienes **71** into arylazo-diazocyclopentadienes **72** was documented [255]. Such products can spontaneously transform into 2-aryltetrazines **73** via reversible electrocyclisation (Scheme 27a). The same reaction can proceed with MeLi and subsequent TsN_3_-associated diazoperoxide transfer to give stable products **75** (Scheme 27b).

**Scheme 28:**
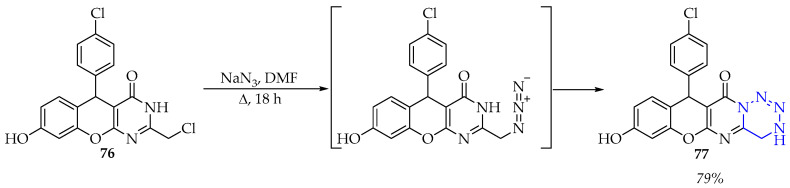
Reaction of 3*H*-chromeno [2,3-*d*]pyrimidine with sodium azide [256].

Another approach to the formation of the 1,2,3,4-tetrazine ring **77** is shown in Scheme 28. This approach is based on the azidation of compound **76**, followed by the intramolecular cyclisation of the azide group with the pyrimidinone nitrogen atom [256].

#### 3.3.2. Synthesis of 1,2,3,5-Tetrazine Derivatives

**Scheme 29:**
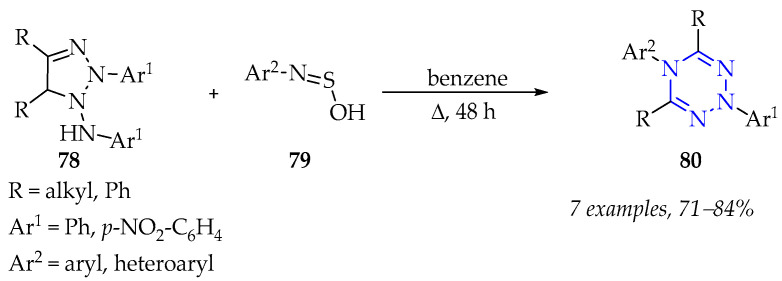
Cycloadditions of aryl-*N*-sulphinylamines with substituted triazolium imides [258].

1,2,3,5-Tetrazines are mainly obtained by the reactions of pyrazoles or triazoles with amines [157,257]. For instance, cycloaddition of aryl-*N*-sulfinylamines **79** to substituted triazolimides **78** gave rise to tetrasubstituted-1,2,3,5-tetrazines **80** (Scheme 29) [258].

**Scheme 30:**
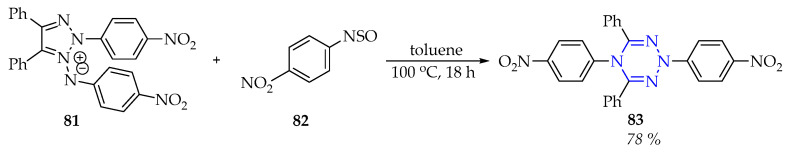
Synthesis of a monocyclic aromatic 1,2,3,5-tetrazine [259].

Substituted 1,2,3,5-tetrazine **83** was prepared by a similar reaction from 1,2,3-triazole **81** and ((4-nitrophenyl)imino)-14-sulfanone **82** (Scheme 30) [259].

**Scheme 31:**
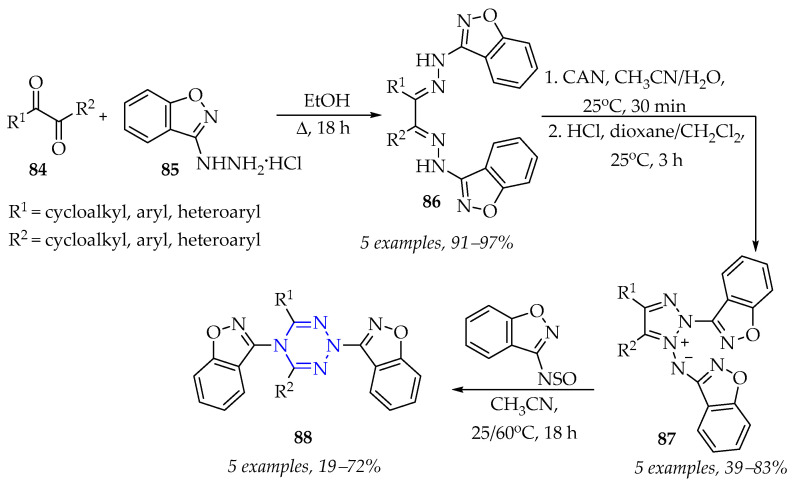
Synthesis of 1,2,3,5-tetrazines [157].

The synthesis can be started with the condensation of 1,2-benzisoxazole-3-hydrazine **85** with the α-diketones **84** to provide the bishydrazones **86** in near quantitative yield. A subsequent (CAN)-mediated oxidation followed by an acid promoted ring-closure reaction generated the zwitterionic *N1,N2*-substituted 1,2,3-triazolium intermediates **87**. Conversion to the dihydro-1,2,3,5-tetrazine system **88** was accomplished through a 1,3-dipolar cycloaddition-triggered rearrangement cascade (Scheme 31).

#### 3.3.3. Synthesis of 1,2,4,5-Tetrazine Derivatives

**Scheme 32:**
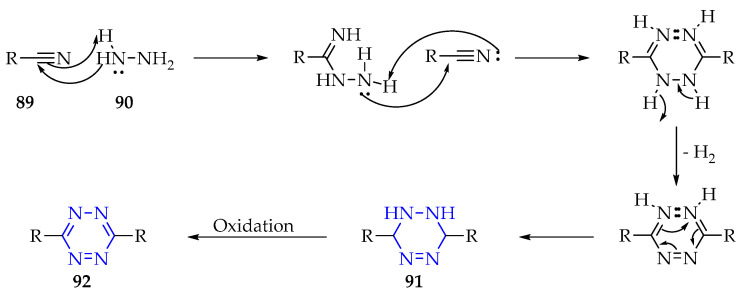
Pinner’s synthesis mechanism [264].

The most studied of the tetrazine derivatives is 1,2,4,5-tetrazine, which is the subject of several reviews [260,261]. Most publications are devoted to the synthesis of both symmetric and asymmetric 3,6-disubstituted *s*-tetrazines, which are assembled by classical methods via the reactions of nitriles or carbaldehydes with hydrazine [262,263]. Pinner’s synthesis plays an important role in *s*-tetrazine chemistry. The dihydro-*s*-tetrazines synthesis involves the reaction of aromatic nitrile **89** with hydrazine **90**, followed by the addition of a second nitrile molecule to give dihydro-*s*-tetrazines **91**, which are further oxidised to *s*-tetrazines **92** (Scheme 32) [264].

**Scheme 33:**
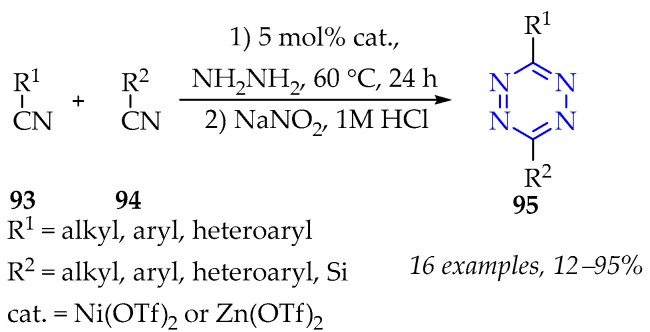
One-pot catalysed synthesis of 1,2,4,5-tetrazines directly from nitriles [265].

Devaraj et al. [265] reported a one-pot synthesis of *s*-tetrazines **95** from aliphatic nitriles **93**, **94,** and hydrazine using metal (Ni and Zn) triphthalates as catalysts (Scheme 33). Zinc salts gave higher yields for less active nitriles such as those that were sterically hindered or affected by electron-donating groups. On the other hand, more reactive nitriles benefited from the use of nickel salts.

**Scheme 34:**
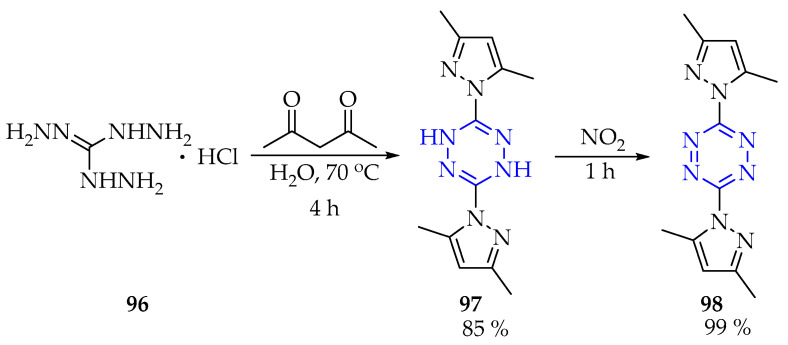
Synthesis of *s*-tetrazines [266].

The work of Ott’s team [266] is of great importance for the synthesis of *s*-tetrazines. This group developed a facile method for the preparation of these compounds involving the treatment of triaminoguanidine monohydrochloride **96** with 2,4-pentanedione to deliver 3,6-bis(3,5-dimethylpyrazol-1-yl)-1,2-dihydro-1,2,4,5-tetrazine **97** in 80–85% yield, followed by oxidation of **97** with nitric oxide to 3,6-bis(3,5-dimethylpyrazol-1-yl)-1,2,4,5-tetrazine **98** (Scheme 34).

**Scheme 35:**
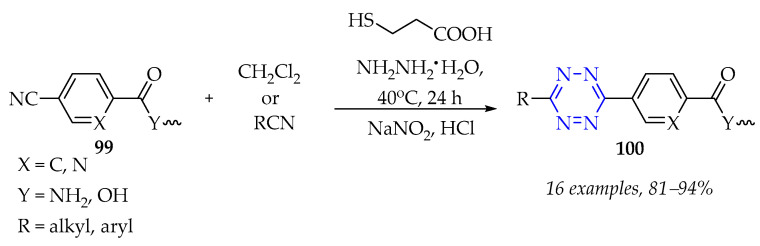
Solid-phase synthesis of *s*-tetrazines [267].

Disubstituted asymmetric aryl- or alkyltetrazines **100** were synthesised by a solid-phase method using readily available nitriles **99** [267] as the starting materials (Scheme 35). This synthetic route was compatible with different solid-phase resins and linkers and did not require metal catalysts or high temperatures.

**Scheme 36:**
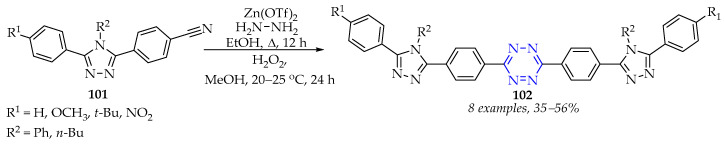
Two-step synthesis of conjugated *s*-tetrazine derivatives [268].

The synthesis of 1,2,4,5-tetrazine derivatives **102** conjugated via a 1,4-phenylene linker to the 4*H*-1,2,4-triazole ring **101** (Scheme 36) [268] was described. This approach leads to the desired products in satisfactory yields, regardless of the nature of the substituents attached to the terminal rings, as well as the type of groups on the triazole nitrogen atom.

**Scheme 37:**
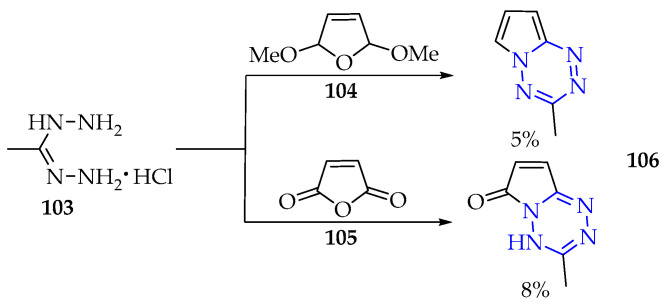
Synthesis of pyrrolo[1,2-*b*][1,2,4,5]tetrazine [269,270,271].

Neunhoeffer’s group has published works describing the synthesis of pyrrolo[1,2-*b*][1,2,4,5]tetrazine derivatives **106**. These were obtained by cyclisation of hydrazidine hydrochloride **103** with 2,5-dimethoxy-2,5-dihydrofuran **104** and maleic anhydride **105** (Scheme 37). The aromatisation of the dihydro derivatives can be achieved through oxidation with manganese dioxide or through hydrogen halide abstraction under the action of triethylamine. Analogous cyclisation of hydrazines with 2-formylbenzoic acid gave similar products **106** [269,270,271].

**Scheme 38:**
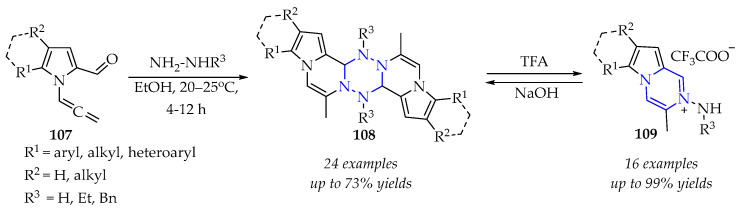
Assembly of fused pyrrolopyrazinotetrazine ensembles and reversible fragmentation [272].

Previously, we reported on the interaction of *N*-allylenylpyrrol-2-carbaldehydes **107** with hydrazine hydrate and substituted hydrazines, which resulted in the assembly of complex pyrrolopyrazinotetrazine ensembles **108** via the formation of aminopyrrolopyrazinium salts **109** (Scheme 38). The syntheses of similar annulated tetrazines were also described in this work [272].

## 4. Conclusions

The review systematises and analyses the possible applications and methods for the synthesis of polyazo six-membered aromatic cyclic frameworks. Despite the large number of examples available in the literature, it is shown that the assembly of such structures usually begins with the interaction of aldehydes, ketones, amines, and/or with molecules containing double or triple bonds with *N*-nucleophiles and is carried out via sequential or intramolecular cyclisation, as well as through the recycling of some nitrogen-containing heterocycles. Each of the described techniques has certain advantages and shortcomings. The latter include the fact that many reactions under consideration require expensive catalysts, long reaction times, are carried out in several stages, etc. Therefore, it is not surprising that the development of more efficient approaches to the synthesis of polyazo heterocycles is an urgent task in modern organic chemistry. Although the list of sources is not exhaustive, the analysis performed indicates that the majority of diazines, triazines, and tetrazines exist in the form of aromatic structures. At the same time, methodologies for the creation of frameworks lacking aromaticity and containing saturated and/or partially saturated polyazo heterocycles are also known. These methodologies will be discussed in the next part of the review.

## Data Availability

Data are contained within the article.

## Abbreviations

The following abbreviations are used in this manuscript:

Δ	Boiling or reflux
Ac	Acetyl
AIDS	Acquired Immune Deficiency Syndromes.
Ar	Aryl
Bn	Benzyl
Boc	*Tert*-butoxycarbonyl group
Bu	Butyl
C(+)|Ni foam(–)	C(+)—graphite plate (cathode), Ni foam(–)—nickel foam (cathode)
CAN	Ceric(IV) ammonium nitrate
CRF	Corticotropin releasing factor
Cy	Cyclohexyl
DABCO	1,4-Diazabicyclo [2.2.2]octane
DBU	Diazabicycloundecene
DCE	Dichloroethane
DMF	*N,N*-Dimethylformamide
DMSO	Dimethyl sulfoxide
Et	Ethyl
Fur	Furyl
IBX	2-Iodoxybenzoic acid
Hfacac	Hexafluoroacetylacetonate
HFIP	1,1,1,3,3,3-Hexafluoroisopropanol
Hx	Hexyl
LiHMDS	Lithium *bis*(trimethylsilyl)amide
NIS	*N*-Iodosuccinimide
Np	Naphtyl
Me	Metyl
MeCN	Acetonitrile
MS	Molecular sieves
MW	Microwave radiation
OTf	Trifluoromethanesulfonic acid
Ph	Phenyl
PIVA	Bis(*tert*-butylcarbonyloxy)iodobenzene
Pr	Propyl
Py	Pyridinyl
TBAClO_4_	Tetrabutylammonium perchlorate
TBS	*Tert*-butyldimethylsilyl group
TFA	Trifluoroacetic acid
Th	Thienyl
TMS	Trimethylsilyl group
Tol	Tolyl
Ts	Toluenesulfonyl
UV	Ultraviolet radiation
Vin	Vinyl
